# Self-rated health and hospital services use in the Spanish National Health System: a longitudinal study

**DOI:** 10.1186/s12913-015-1158-8

**Published:** 2015-11-04

**Authors:** Nayara Tamayo-Fonseca, Andreu Nolasco, Jose A. Quesada, Pamela Pereyra-Zamora, Inmaculada Melchor, Joaquin Moncho, Julia Calabuig, Carmen Barona

**Affiliations:** Department of Community Nursing, Research Unit for the Analysis of Mortality and Health Statistics, Preventive Medicine, Public Health and History of Science. University of Alicante. Campus de San Vicente del Raspeig s/n, Apartado 99, 03080 Alicante, Spain; Registro de Mortalidad de la Comunidad Valenciana, Servicio de Estudios Epidemiológicos y Estadísticas Sanitarias. Subdirección General de Epidemiología y Vigilancia de la Salud. Conselleria de Sanitat, Plaza de España 6, 03010 Alicante, Spain; Servicio de Análisis de Sistemas de Información Sanitaria, Conselleria de Sanitat, Generalitat Valenciana. C/Micer Mascó, 31-33, 46010 Valencia, Spain; Servicio del Plan de Salud, Dirección General de Salud Pública. Conselleria de Sanitat, Generalitat Valenciana. Avda. Cataluña, 21, 46020 Valencia, Spain

**Keywords:** Self-rated health, Hospitalization, Health services use, Health indicators, Longitudinal study

## Abstract

**Background:**

Self-rated health is a subjective measure that has been related to indicators such as mortality, morbidity, functional capacity, and the use of health services. In Spain, there are few longitudinal studies associating self-rated health with hospital services use. The purpose of this study is to analyze the association between self-rated health and socioeconomic, demographic, and health variables, and the use of hospital services among the general population in the Region of Valencia, Spain.

**Methods:**

Longitudinal study of 5,275 adults who were included in the 2005 Region of Valencia Health Survey and linked to the Minimum Hospital Data Set between 2006 and 2009. Logistic regression models were used to calculate the odds ratios between use of hospital services and self-rated health, sex, age, educational level, employment status, income, country of birth, chronic conditions, disability and previous use of hospital services.

**Results:**

By the end of a 4-year follow-up period, 1,184 participants (22.4 %) had used hospital services. Use of hospital services was associated with poor self-rated health among both men and women. In men, it was also associated with unemployment, low income, and the presence of a chronic disease. In women, it was associated with low educational level, the presence of a disability, previous hospital services use, and the presence of chronic disease. Interactions were detected between self-rated health and chronic disease in men and between self-rated health and educational level in women.

**Conclusions:**

Self-rated health acts as a predictor of hospital services use. Various health and socioeconomic variables provide additional predictive capacity. Interactions were detected between self-rated health and other variables that may reflect different complex predictive models, by gender.

## Background

Self-rated health (SRH) is a simple measure that has been shown to be a good indicator since it provides a global overview of a population’s health status and has been related at the individual level to indicators such as mortality, morbidity, functional capacity, and use of health services, among others [1–4]. This subjective measure has been related to people’s willingness to make an effort to maintain a good state of health [5].

As several studies have indicated, one of the advantages of assessing self-rated health is that this measure incorporates individual perspectives on health and disease. Nevertheless, there is widespread debate about its use in public health policies because it has been suggested that self-rated health may provide misleading information or could underestimate social inequalities in health [6]. However, recent research findings reported in several studies have indicated that the predictive validity of SRH has increased over time [7].

Previous studies, mostly conducted in the United States and Finland, have demonstrated that self-rated health is a predictor of health services use [1, 3, 8–12], as well as a good indicator of the health and medical care needs and services in a community [13]. It has been suggested that monitoring SRH could constitute a practical tool for health managers, researchers and policymakers when assessing possible changes in health service needs [9], as well as for predicting hospital admission [12] and identifying older adults at increased risk for admission to nursing homes [8]. In addition, this indicator has been used to identify groups at risk so as to analyze hospital costs [10, 12].

It has been reported that health status is a major determinant of the use of hospital services (HS) in universal health care systems [14]. Thus, there is a need for indicators that make it possible to monitor changes in a population’s health status, the system’s responses and consequently, health outcomes. Objective measures such as annual number of visits to the doctor, average hospital stay, or number of beds have been widely used in studies on health services use. Self-rated health is a short measure, with the advantage of being less expensive and less burdensome to collect, and can conceivably be collected with relative ease [3].

As a result of European Union recommendations for improving information systems, those responsible for making decisions on health currently use various harmonized resources as their main sources of information, such as health surveys and medical and administrative records, which together provide an accessible and updated overview of changes in health status and available resources.

Some studies have examined the association between SRH and HS use. However, this relationship has not yet been clearly established in Spain, partly because of the difficulties involved in associating this type of indicator with different sources of information, and partly because SRH has only been studied in elderly populations [15–17]. A previous study conducted in the Region of Valencia (RV) linked the 2005 Region of Valencia Health Survey (RVHS2005) cohort to the RV mortality register; after 4 years of monitoring, it was found that SRH acted as a predictor of mortality in the general population for both men and women, and also mediated between socioeconomic, demographic, and health conditions, and mortality [18]. At national level, however, few studies in Spain [16, 19] have attempted to link a cohort to information derived from the Minimum Basic Hospital Data Set (MBDS) so as to establish profiles on the use of HS and hospitalization outcomes.

Given the lack of longitudinal studies in Spain associating SRH with the use of HS in the general population, the aim of the present study was to describe and analyze the association and predictive capacity of SRH, socioeconomic, demographic, and health status variables as regards HS use in people over 20 years of age, based on monitoring the RVHS2005 adult cohort linked to the MBDS of public hospitals in the Region of Valencia.

## Methods

### Study design and sample

This is a longitudinal follow-up study of a cohort of adults who comprised the RVHS2005 sample [20]. The initial sample consisted of 5,481 participants, all of whom were aged over 20 years and were noninstitutional residents of the RV, a Mediterranean region in eastern Spain (5,004,844 inhabitants in 2005). The study sample was representative of the RV and was obtained by means of a complex sample design whereby all participants were assigned a weight according to their representativeness, and these weights were included in the RVHS2005 databases supplied by the Health Plan Office at the Health Department of the Regional Government of Valencia.

### Measures

The study’s response variable was use of HS, dichotomized into “use”/”non-use”. Use was defined as any admission to the hospital for acute care or major outpatient surgery (including admissions with a stay less than 24 h) and chronic or long-stay hospitalization.

At the time of this study, the overall population in Spain had health insurance and medical coverage, and a physician decision was needed for hospitalization, which was scheduled or carried out through the emergency service. Hospitalization related to childbirth and caesarean section (180 cases, 6.8 % of admissions) was excluded. Results for the response variable were obtained by means of linking the RVHS2005 database to the Hospital Discharge Register (the MBDS) for 32 hospitals in the RV, over a 4-year follow-up period from 1 January 2006 to 31 December 2009. It is mandatory for all public hospitals in Spain to update the MBDS, which aggregates all information from hospitals in a standardized, coded, electronic format that is compatible with other information systems. The register is also used by various researchers and planners as a source of data for analysis of health status and outcomes, as well as for health planning and resource assessment [19, 21–24].

The main explanatory variable was SRH, based on the following RVHS2005 question: “Right now, how would you describe your health?” In accordance with common recoding practice, the possible answers (Very good/Good/Fair/Poor/Very poor/Don’t know or no answer) were aggregated into two categories: Good (good or very good) and Poor (fair, poor or very poor). To control for the possible explanatory or confounding effect or interaction of the socioeconomic context and morbidity, the following explanatory variables with their corresponding categories were included in the analyses: sex (female, male), age (21–44, 45–64, 65–84, ≥ 85 years), educational level (primary or lower, higher than primary), employment status (working, not working), self-rated income level (low, medium-high), country of birth (Spain, abroad), presence of chronic disease (yes, no) or disability (yes, no), and use of HS in the year prior to the survey (yes, no). Categorization of the variables was made according to a previous study on SRH and mortality using this cohort [18]. The question addressing self-rated income level was “How would you describe your level of income?” Possible responses were: High/Medium–high/Medium/Medium–low/Low/Don’t know, or no answer provided. This variable was recategorized into Low (low or medium–low) and Medium–high (high, medium–high or medium).

### Data analysis

Frequencies and percentages of HS use were calculated for each level of the explanatory variables, by sex and for the total. To analyze the association between use of HS and the explanatory variables, logistic regression models were adjusted with the response variable (use of HS; yes/no), using the likelihood ratio to test likelihood increases between models, and the Wald statistic to test significance for the variables included in each model. The odds ratio (OR) was calculated as a measure of the association between use of HS and the explanatory variable categories, together with the corresponding 95 % confidence intervals (95 % CI). ORs were obtained for HS use with each explanatory variable in a simple analysis, adjusted for age, and finally adjusting for all variables, including and removing SRH to determine its effect on the model. An analysis was conducted of the possible interaction between SRH as the main explanatory variable and the other explanatory variables. All analyses were performed separately by sex and considering the complex sample design. The weights of the sample participants were introduced in the weights option of the statistical package. Statistical analysis was performed using the R 2.12.2 software package (R Foundation for Statistical Computing, Vienna, Austria).

### Ethical considerations

Owing to the characteristics of this prospective observational study based on administrative data, this research posed no ethical issues because the researchers only had access to data rendered anonymous via a complex process of linking databases, which were only handled by authorized staff from the responsible information systems departments of the Region of Valencia Department of Health.

## Results

A total of 206 participants were excluded from the follow-up, either because their records could not be linked with complete certainty (178 cases) or because they had died in 2005 (28 cases), leaving a total of 5,275 participants for follow-up and analysis.

During the period monitored, 1,184 (22.4 %) individuals used HS, of whom 585 were men (49.4 %) and 599 women (50.6 %). No significant difference by sex was observed in the percentage of HS use, which was 22.6 % for men and 22.3 % for women. Table 1 shows the number and percentage of individuals who used HS, separated by sex and for the total, for each level of the explanatory variables. For men, women, and the total alike, greater use was made of HS by participants who reported poor self-rated health, chronic disease, or disability, those who had a primary education or lower, low income, and who were older, unemployed, born in Spain, and who had used HS in the previous year.

**Table 1:** Frequency and percentage distributions of hospital service use by sex, according to explanatory variable categories

Explanatory variables (missing data^a^)		Use of hospital services					
		Men		Women		Total	
		n	%	n	%	n	%
Total sample		585	22.6	599	22.3	1184	22.4
Self-rated health (18)	Good	372	18.4	317	17.0	689	17.7
	Poor	211	37.5	279	34.7	490	35.9
Age (9)	21-44	191	14.0	200	15.4	391	14.7
	45-64	175	22.5	161	20.7	336	21.6
	65-84	207	48.8	203	38.2	410	43.0
	85+	12	46.2	33	46.5	45	46.4
Employment status (168)	Working	274	15.6	173	16.3	447	15.8
	Not working	286	37.9	386	25.3	672	29.5
Educational level (75)	Primary or lower	419	26.6	444	26.0	863	26.3
	Higher than primary	158	16.1	149	15.8	307	16.0
Income level (505)	Medium-high	324	20.1	307	19.7	631	19.9
	Low	216	29.2	235	27.4	451	28.2
Country of birth (284)	Spain	523	23.7	510	22.4	1033	23.0
	Other	36	14.5	43	17.1	79	15.8
Chronic disease (4)	Yes	391	28.6	480	26.8	871	27.6
	No	195	15.8	118	13.3	313	14.8
Disability (7)	Yes	86	38.7	111	41.9	197	40.5
	No	498	21.0	487	20.2	985	20.6
Previous use of hospital services (11)	Yes	303	26.7	366	25.9	669	26.3
	No	282	19.3	232	18.3	514	18.9

^a^Total number of people who responded “Don’t know” or did not provide an answer

A simple analysis revealed that all variables were significant predictors of HS use in the follow-up period for both men and women, except in the case of the country of birth variable among women (Table 2 and Table 3). When adjusted for age, all variables except disability were significant predictors for men, whereas significant predictors for women were poor self-rated health, chronic disease, disability, and previous use of HS.

**Table 2:** Association between use of hospital services and studied variables, among men

Men		Simple analysis^a^	Adjusted by age^a^	Analysis adjusting for all variables, not including SRH^b^ L^c^ = 206.6; df = 6	Analysis adjusting for all variables, including SRH^b^ L^c^ = 219.9; df = 8
Age	21–44	1	-	1	1
	45–64	**1.8** (1.4–2.2)		**1.5** (1.2–1.9)	**1.4** (1.1–1.8)
	65–84	**5.9** (4.6–7.6)		**3.5** (2.4–5.1)	**3.4** (2.3–5.0)
	85+	**5.3** (2.4–11.8)		**3.1** (1.3–7.4)	**3.0** (1.2–7.3)
Self-rated health	Good	1	1		1
	Poor	**2.7** (2.2–3.3)	**1.8** (1.4–2.2)		0.7 (0.3–1.5)
Chronic disease	No	1	1	1	1
	Yes	**2.1** (1.7–2.6)	**1.3** (1.1–1.6)	**1.3** (1.1–1.7)	1.1 (0.8–1.4)
Disability	No	1	1	NS	NS
	Yes	**2.4** (1.8–3.2)	1.4 (0.9–1.9)		
Employment status	Working	1	1	1	1
	Not working	**3.3** (2.7–4.1)	**1.5** (1.1–2.0)	**1.5** (1.1.–2.0)	**1.4** (1.0–1.9)
Educational Level	Higher than primary	1	1	NS	NS
	Primary or lower	**1.9** (1.5–2.3)	**1.3** (1.0–1.6)		
Income level	Medium-high	1	1	1	1
	Low	**1.6** (1.3–2.0)	**1.3** (1.1–1.6)	**1.3** (1.0–1.6)	**1.3** (1.0–1.6)
Previous use of hospital services	Yes	1	1	NS	NS
	No	**1.5** (1.3–1.8)	**1.2** (1.0–1.5)		
Country of birth	Other	1	1	NS	NS
	Spain	**1.9** (1.3–2.7)	**1.5** (1.0–2.2)		
Chronic disease NO	Good health	-	-		1
	Poor health				0.7 (0.3–1.5)
Chronic disease YES	Good health				1
	Poor health				**1.7** (1.3–2.5)

^a^Odds ratios (95 % confidence interval). Statistically significant odds ratios in bold, *P* <0.05

^b^Final model included only variables with significant adjusted effects

^c^ *L* Likelihood ratio test for assessing significance of likelihood increase for the model vs. the model with only a constant

Abbreviations: *SRH* self-rated health; df, degrees of freedom, *NS* not significant

**Table 3:** Association between use of hospital services and studied variables, among women

Women		Simple analysis^a^	Adjusted by age^a^	Analysis adjusting for all variables, not including SRH^a,b^ L^c^ = 177.7; df = 7	Analysis adjusting for all variables, including SRH^a,b^ L^c^ = 198.3; df = 9
Age	21–44	1	-	1	1
	45–64	**1.4** (1.2–1.8)		**1.2** (0.9–1.5)	1.1 (0.8–1.4)
	65–84	**3.4** (2.7–4.2)		**2.5** (1.9–3.2)	**2.2** (1.7–2.9)
	85+	**4.8** (3.0–7.8)		**3.4** (2.0–5.7)	**2.7** (1.6–4.6)
Self-rated health	Good	1	1	-	1
	Poor	**2.6** (2.2–3.1)	**1.9** (1.5–2.3)		**2.6** (1.7–4.0)
Chronic disease	No	1	1	1	1
	Yes	**2.4** (1.9–3.0)	**1.8** (1.4–2.2)	**1.6** (1.3–2.0)	**1.4** (1.1–1.8)
Disability	No	1	1	1	1
	Yes	**2.9** (2.2–3.7)	**1.8** (1.3–2.4)	**1.6** (1.2–2.1)	**1.5** (1.1–2.0)
Employment status	Working	1	1	NS	NS
	Not working	**1.7** (1.4–2.1)	1.0 (0.8–1.3)		
Educational Level	Higher than primary	1	1	1.2 (0.9–1.5)	1
	Primary or lower	**1.9** (1.5–2.3)	1.2 (0.9–1.5)		**1.4** (1.0–1.8)
Income level	Medium-high	1	1	NS	NS
	Low	**1.5** (1.3–1.9)	1.2 (0.9–1.4)		
Previous use of hospital services	Yes	1	1	1	1
	No	**1.6** (1.3–1.9)	**1.5** (1.2–1.8)	**1.3** (1.1–1.6)	**1.3** (1.1–1.6)
Country of birth	Other	1	1	NS	NS
	Spain	1.4 (1.0–2.0)	1.2 (0.8–1.6)		
Educational level: Higher than primary	Good health	-	-	-	1
	Poor health				**2.6** (1.7–4.0)
Educational level: Primary or lower	Good health				1
	Poor health				**1.3** (1.0–1.5)

^a^Odds ratios (95 % confidence interval). Statistically significant odds ratios in bold, *P* <0.05

^b^Final model included only variables with significant adjusted effects

^c^ *L* Likelihood ratio test for assessing significance of likelihood increase for the model vs. the model with only a constant

Abbreviations: *SRH* self-rated health, *df* degrees of freedom, *NS* not significant

In the model adjusted for all variables except SRH, those participants most at risk of hospitalization were unemployed men with low income and chronic disease. When SRH was included in the model, a significant interaction was seen between SRH and the presence of chronic disease, significantly increasing the log likelihood ratio from 206.6, with 6 degrees of freedom to 219.9, with 8 degrees of freedom (*p* < 0.001). Thus, in the group of people with chronic disease, those who reported poor health were significantly more likely to be hospitalized than those reporting good health (OR = 1.7, 95 % CI = [1.3–2.5]). However, the result was not significant in the group without chronic disease (OR = 0.7, 95 % CI = [0.3–1.5]).

When adjusted for all variables except SRH, having chronic disease, disability or previous use of HS significantly increased the likelihood of using HS for women. When SRH was included in the model, these three variables remained significant, and a significant interaction between SRH and educational level was also observed, significantly increasing the log likelihood ratio from 177.7, with 7 degrees of freedom to 198.3, with 9 degrees of freedom (*p* < 0.001). Thus, those who reported poor health were more likely to be hospitalized, but the association was significantly stronger in the group with higher educational level (OR = 2.6, 95 % CI = [1.7–4.0]) than in the group with lower educational level (OR = 1.3, 95 % CI = [1.0–1.5]).

## Discussion

This study revealed that in Spain, SRH is a predictor of HS use. After 4 years of following the RVHS2005 cohort, participants who reported poor SRH had a higher risk of using HS than people who reported good health. This predictive role was significant even after adjusting for age, health status, and sociodemographic characteristics.

These findings are consistent with those reported in studies conducted in other countries, in which SRH has been described as one of the determinants of hospitalization and the use of other health services. For example, De Salvo et al. [3] found that poor SRH was a predictor of mortality, hospitalization, and high use of outpatient services. A study by Weinberger et al. [8] showed that poor SRH was a risk factor for hospitalization and residence in nursing homes or homes for the elderly. McGee et al. [11] found that people with poor SRH presented higher values for two measures of HS use, namely, mean number of days in bed and average number of visits to the doctor in the previous 12 months; these results are similar to findings reported in a study by Miilumpalo et al. [9] conducted in Finland.

In this study, we found that over the 4 years of follow-up, 22.4 % of respondents used HS. Using a follow-up period of the same length but in a cohort aged over 64, Suárez García [17] obtained a higher hospitalization percentage (29.6 %) for the overall follow-up, which could be attributed to the older age of the cohort studied. In our study, the mean annual percentage obtained for hospitalization was 5.6 %. This figure is lower than that reported in other cross-sectional studies [25, 26], in which the annual percentage obtained for hospital admissions in Spain was 9.3 %, a little lower than the mean of 10.9 % reported for the European Union [27]; however, these studies were based on self-reported responses, which may explain the differences. Differences could be also explained by the exclusion of childbirths and caesarean sections in our study.

According to results of the simple analyses, the most common profile of patients who had used HS was that of participants who reported poor self-rated health, were older, born in Spain, unemployed, had primary education or below, a low income, chronic disease, disability, or had used health services in the previous year. These results are consistent with findings obtained in other studies conducted in Spain, although there are some differences.

With regard to age, a sharp increase in hospital admissions in Spain in recent years has been reported, which is partially attributable to the aging population [14]. However, another study has suggested that use of hospital resources may not be related to age so much as to the type of hospital admission [28]. In that study as well as in the majority of studies [29], no significant differences were found by sex. However, some studies in Spain have reported sex inequalities in the provision of hospital care that favor men [14–16, 30, 31], which highlights important implications for healthcare organizations. It is reasonable to assume that had diagnosis codes related to childbirth been included in this study, we would likely have found a slight predominance of HS use by women; such a finding would coincide with the results of the hospital register [32] as well as with recent data from cross-sectional studies, the Spanish Health Survey [26], and the Region of Valencia Health Survey [25].

Our results for country of birth agree with those of the last Spanish Health Survey (2011–12), indicating that Spanish-born people make most use of HS. However, some studies differ in this respect, perhaps because of the heterogeneity of the groups analyzed. These studies have reported a greater tendency among immigrants to use hospital emergency services as a substitute for other healthcare services [33, 34], although there is evidence that utilization rates among Spanish people of outpatient, emergency, and hospitalization services exceed the corresponding rates among immigrants [35, 36]. Our results regarding lower use by immigrants could be explained by the exclusion of diagnoses related to childbirths, which are more common among immigrant women [35]; possible difficulties for immigrants to access the health system [37]; and the “healthy migrant effect” that has been described in Spain [38].

With regard to education, we found that a low educational level was associated with increased use of HS. This result coincides with the trend observed in health surveys in Spain and the Region of Valencia, in which people who could not read or had not completed their primary education presented higher utilization rates than those with tertiary or vocational education, although with slight variations by age and sex [25, 26]. However, our result differs from that of another study in which the opposite association was observed, namely, that people with a higher educational level tended to consume more health services, owing in part to the attitude of doctors regarding the degree of patient information [39].

A multivariate analysis adjusting for all the variables studied revealed the importance for men of socioeconomic variables (employment status and income level) and the interaction of SRH with chronic disease. In contrast, the most important variables for women were those related to health status (chronic disease and disability) and previous use of HS, as well as the interaction of SRH with educational level.

Although this study only analyzed data pertaining to the public health system and thus excluded private hospitals, which provide HS to a higher percentage of high income individuals [40], the adjusted results for men nevertheless indicate that independently of SRH, income level was a predictor of HS use. Other studies have reported finding no significant inequalities by socioeconomic status for hospitalization in Spain [14, 40–42]. In agreement with findings reported by Regidor et al. [43] and the Region of Valencia [25], our results show that people with lower incomes presented higher rates of hospitalization.

The results of both simple and multivariate analyses of employment status indicate that independently of SRH, unemployed men were at the highest risk of hospitalization. The lower rates of HS use among employed people might in part be owing to the higher time/opportunity costs faced by working individuals, which are reflected in their greater reluctance to initiate or prolong a stay in hospital [39].

For both sexes, the presence of chronic disease increased the risk of HS use. This result agrees with those of previous studies because chronic disease, degree of severity, and comorbidity have all been described as important risk factors for hospitalization [16, 17, 29, 30, 44].

For men, the interaction between SRH and the presence of chronic disease led to increased risk of hospitalization when poor SRH was reported, but only among those who had a chronic disease. This result might indicate that men with chronic disease who reported poor SRH reinforced their negative assessment owing to poor health status, which probably led them to be hospitalized. This hypothesis might correspond to the relationship between SRH and mortality described by other authors, whereby men but not women who rated their health as poor were found to be at higher risk of dying, suggesting that men with chronic disease had more life-threatening conditions whereas women had more disabling conditions, and consequently made less use of HS and greater use of primary healthcare [18, 30, 45].

The results for women show that independently of SRH, previous use of HS was a predictor of HS use, in agreement with other studies in which this variable has been described as a major predictor of subsequent hospitalization and has also been associated with increased mortality and stay in nursing homes [15]. Disability and chronic disease were also predictors of HS use, again independently of SRH, and these have also been described as predictors of mortality [18, 45].

Educational level presented a complex behavior among women, interacting with SRH in such a way that risk of HS use was higher among women reporting poor SRH; however, the association was stronger in those with higher educational level than in those with lower educational level. This latter finding may be consistent with the effect described by Schnittker and Bacak [7], whereby the predictive validity of SRH increases as educational level rises. This finding may also be in line with other studies that have analyzed the role of education in the search for and consumption of health services [39, 41] and in which several hypotheses have been proposed, for instance that the use of health services depends on the degree to which individuals value their own health, and thus efficient production of health increases as educational level rises. Another hypothesis proposes that people with a higher educational level tend to use health services more often and in a more timely manner, owing in part to a greater awareness about the importance of health and different attitudes towards the health system, leading to a broader demand for healthcare and better knowledge of how the health system functions.

The end of the study period coincided with the beginning of the severe economic downturn that has been affecting Europe and Spain since 2008, and our results correspond to a period of public and universal healthcare in Spain. Although Spain’s universal healthcare system should ensure equitable access, several studies have explored the existence of inequalities in use of the public healthcare system [39–41, 46], which may have increased in recent years as a consequence of changes in the economic context and health and social reforms [47, 48].

These inequalities may partially explain some of the results obtained in our study. Changes to the healthcare model such as restrictions on certain health benefits, limiting the basic portfolio of services, exclusion or limitation of various groups (for instance, undocumented immigrants who were previously entitled to receive certain medical treatments), or implementation of co-payment, should be avoided since they could widen disparities in primary and hospital healthcare service use, as has been reported in other European countries with policies aimed at reducing public spending on health [49].

The expected changes in health outcomes as a consequence of changes resulting from the economic crisis suggest the need to monitor indicators such as SRH, together with socioeconomic and health indicators, such as those used in this study.

### Strengths and limitations

One of the strengths of this study was the size of the study sample and its representativeness relative to the general population. Moreover, the study included the general population aged over 20 years old, encompassing a wide age range but adjusting for age in the models. Another strength of the study was its use of data from the RVHS2005, which was designed and validated for obtaining population data on the variables studied and had a very high response rate.

Limitations included losses during follow-up, respondents who may have used HS in other locations or people who had used private hospitals, since it has been reported that people with a more favorable socioeconomic status are more likely to use additional private insurance [40, 41, 46]. In addition, the exclusion of participants who died in 2005 may have reduced the impact of the hospitalization factors studied; these participants might have had worse health indicators [16] but could not make use of HS because they had died.

Regarding the quality of data from the MBDS, this information system is now widely used in clinical and epidemiological research, and although initially some studies had reported various limitations concerning the quality of clinical information [22, 50] others have since established its reliability and validity as a source for observational study [23, 24].

One of the most widely used indicators of HS use is volume of use, which includes number of admissions and length of stay. Our study is based only on the use or non-use of HS, without considering high-frequency users. Length of stay and total number of admissions might present different results.

## Conclusions

This study has shown that SRH is a predictor of hospital service use after 4 years of follow-up among the general population in Spain, a subject which has received little research attention in that country. SRH can be considered an indicator that mediates between the socioeconomic and health status of the population and use of HS, although consideration of other variables (chronic disease, disability, educational level, employment status, income level, or previous use of services) improves predictive capacity regarding HS use. For men, greater risk of HS use was explained by socioeconomic variables and chronic disease, whereas for women it was explained by health status, educational level, and previous use of services.

The interactions detected may reflect complex models. For men, poor SRH was predictive of HS use when combined with chronic disease, whereas for women it was predictive when combined with any educational level, but more intensely at the highest level.

This study suggests that in the current context of economic instability, efforts must be made to maintain social and health policies and employment protection because these exert a positive influence on the health of the population and on use of health services. It is necessary to monitor the self-rated health and socioeconomic and health status of the population.

Given the paucity of studies in Spain that have assessed the predictive capacity of SRH for HS use, and given recent socioeconomic changes, further research is necessary on this subject.

## Abbreviations

CI: Confidence interval
HS: Hospital services
MBDS: Minimum basic hospital data set
OR: Odds ratio
RV: Region of Valencia
RVHS2005: Region of Valencia Health Survey 2005
SRH: Self-rated health
